# Fc-modification of anti-PcrV gene-encoded antibodies modulates complement-mediated killing of Pseudomonas aeruginosa

**DOI:** 10.3389/fimmu.2025.1618297

**Published:** 2025-07-31

**Authors:** Jillian Eisenhauer, Spencer Dublin, Jihae Choi, Abigail R. Trachtman, Jacqueline D. Chu, David Custodio-Zegarra, Suman Bharti, Bhavya Bhardwaj, Shuangyi Bai, William T. Witt, Maria de la Paz Gutierrez, Sarah J. Miller, Kaitlyn Flowers, Trevor R. F. Smith, Bronwyn M. Gunn, Mariette Barbier, Elizabeth M. Parzych, David B. Weiner, Ami Patel

**Affiliations:** ^1^ Perelman School of Medicine University of Pennsylvania, Philadelphia, PA, United States; ^2^ Vaccine & Immunotherapy Center, The Wistar Institute of Anatomy and Biology, Philadelphia, PA, United States; ^3^ Department of Microbiology, Immunology, and Cell Biology, West Virginia University, Morgantown, WV, United States; ^4^ Paul G. Allen School for Global Health, College of Veterinary Medicine, Washington State University, Pullman, WA, United States; ^5^ Inovio Pharmaceuticals, Plymouth Meeting, PA, United States

**Keywords:** *Pseudomonas aeruginosa*, PcrV, complement system, DNA-encoded monoclonal antibody, type III secretion system

## Abstract

*Pseudomonas aeruginosa* is a high priority multi-drug-resistant (MDR) bacterial pathogen with increasing resistance against broad-spectrum antibiotics. Multiple efforts are ongoing to develop anti-pseudomonal vaccines however achieving meaningful outcomes has been challenging in human clinical trials. Monoclonal antibodies (MAbs) are emerging as promising biologics for targeting *P. aeruginosa* infections and engineering strategies that bridge engagement with innate immune mechanisms like complement-mediated antibody dependent phagocytosis may be beneficial to improve bacterial clearance. We previously described both protection and long-term expression of synthetic DNA-encoded MAb (DMAb) expressing the anti-PcrV MAb V2L2-MD. Here, we show that modification of DMAb-V2L2-MD with an Fc-point mutation designed to enhance complement engagement demonstrates improved binding to C1q, C3 deposition, and improved opsonophagocytic killing. This Fc-modified DMAb reduced *P. aeruginosa* bacteria burden in lungs and nasal washes in a lethal acute murine intranasal infection model. These data highlight the importance of tailoring downstream antibody innate effector functions to improve clearance of difficult-to-treat bacteria like MDR *P. aeruginosa.*

## Introduction

Antimicrobial resistance (AMR) is a major public health threat, with an increasing number of bacterial pathogens that are developing multi-drug resistance, including several resistant to last-resort antibiotics. In 2019, there was an estimated 4.95 million deaths associated with bacterial AMR and 1.27 million deaths attributable to bacterial AMR, globally (1). It is now estimated that by 2050 there will be a significant increase of 10 million deaths per year attributable by AMR pathogens and become the world’s primary cause of death if no intervention is taken (2, 3).

*Pseudomonas aeruginosa* is a multi-drug resistant (MDR) opportunistic bacterium and one of the main causative agents of hospital-acquired infections (4), including major resistant ventilator associated pneumonia (VAP). These infections are often responsible for increasing the length of hospital stays (>5 days). Treatment of *P. aeruginosa* infections have become increasingly difficult predominantly due to the bacterium’s rapid mutational rate and a 32% rise in AMR due to overuse of antibiotics during the years of the SARS-CoV-2 pandemic (5, 6). Many of these high-priority pathogens are classified as Group D bacterial pathogens by the World Health Organization. Group D bacterial pathogens, which includes *P. aeruginosa*, are associated with low feasibility of vaccine development due to complicated pathogen biology and critically ill target populations with little time for patients to mount an effective immune response to vaccines (7).

With a lack of new antibiotic therapeutics or successful vaccine candidates, MAb approaches have emerged as a potential strategy for protection against viral and bacterial pathogens, especially for use in high-risk, immunocompromised patients (8–10). One promising MAb candidate, V2L2-MD, targets the PcrV protein of the *P. aeruginosa* Type III Secretion system (T3SS), commonly associated with increased mortality and bacterial virulence (11–13). In a Phase I clinical trial (NCT02255760), bispecific antibody MEDI3902 (anti-PcrV V2L2-MD and anti-Psl) exhibited minimal adverse events and linear pharmacokinetics with a serum concentration of 5 µg/mL through day 28 (14). To build on this work, we previously described *in vivo* delivery of synthetic plasmid DNA-encoded monoclonal antibodies (DMAb) encoding V2L2-MD as additional modality for *in vivo* delivery of protective MAb with potential to enhance bioavailability and global drug biologic accessibility, demonstrating protection by V2L2-MD DMAb in a mouse pneumonia challenge model (15).

However, in a Phase II clinical trial (NCT02696902), a 1500 mg dose of MEDI3902 did not reduce *P. aeruginosa* nosocomial pneumonia in colonized ventilated patients despite reaching protective levels in sera (16–18). Therefore, further development of sequence engineering strategies that improve the potency of MAb has the potential to increase the clinical efficacy of these countermeasures against MDR. *P. aeruginosa.* Recent advances in Fc-engineering of therapeutic antibodies to improve microbial killing and tumor clearance may be of interest (19). Ultimately, the control and clearance of *P. aeruginosa* depends on phagocyte recognition, engulfment, and degradation of bacteria (20). Furthermore, animal model studies highlight a critical role for the complement system as complement deficient mice are less effective in controlling *P. aeruginosa* infections (21–23). The single amino-acid Fc-E430G modification has been shown to modulate antibody effector activity leading to improved complement system activation (24–28). In a vaginal mucosal challenge, anti-gonococcal antibody 2C7-Fc-E430G demonstrated enhanced complement activation, and subsequent downstream membrane attack complex (MAC) formation, which improved bacterial clearance against *Neisseria gonorrhea* compared to wild-type 2C7 IgG1 Fc (29). However, the importance of complement modifications has not been described in anti-*P. aeruginosa* MAbs.

Here, we compared intact Fc V2L2-MD DMAb to an abrogated Fc variant with loss of Fc-effector function, hypothesizing that the addition of Fc-modification E430G can enhance MAb-mediated complement clearance of *P. aeruginosa.* We tested whether V2L2-MD DMAb Fc-variants modulate complement-mediated antibody-dependent cellular phagocytosis (cADCP) and antibody-dependent complement deposition (ADCD) *in vitro.* For *in vivo* studies, we utilized an acute *Pseudomonas aeruginosa* (PAO1) infection in a mouse model. Overall, the Fc-E430G modification improved V2L2-MD DMAb *in vitro* C1q engagement and C3 deposition and demonstrated a significant improvement in *in vivo* bacterial clearance during challenge. Our data show an important role for complement-engagement in MAb-mediated clearance of *P. aeruginosa* in the lung that has significant potential to enhance the potency of recombinant and gene-encoded MAbs against respiratory AMR pathogens and perhaps others.

## Materials and methods

### Ethics Statement

Use of animals was performed in strict accordance with recommendations in the Guide for the Care and Use of Laboratory Animals of the National Institutes of Health (30). Protocols were approved by the Institutional Animal Care and Use Committees (IACUCs) at West Virginia University (protocol 1606003173) and The Wistar Institute (protocol 201124).

### Cell lines and bacteria

Human embryonic kidney (HEK) 293 T cells (ATCC; Cat# CRL-1573) were maintained in Dulbecco’s Modified Eagle’s Medium (ThermoFisher Scientific) supplemented with 10% fetal bovine serum (FBS). Expi293F cells (ThermoFisher Scientific; Cat# A14527) were maintained in Expi293 media as per manufacturer’s instructions (ThermoFisher Scientific; Cat# A1435101). HL60 cells (ATCC; Cat# CCL-240) were maintained in Iscove’s Modified Dulbecco’s Media (IMDM) supplemented with 20% FBS, as suggested by the manufacturer’s instructions. All cell lines were maintained in mycoplasma negative conditions. Routine testing was performed at the University of Pennsylvania. All cells were maintained at a low passage number.

*P. aeruginosa* strain PA14 strain (NR-50573; BEI Resources) was used for all *in vitro* experiments. *P. aeruginosa* strain PAO1 was a gift from Dr. Michael L. Vasil at the University of Colorado and was maintained and used as previously described with minor changes (31, 32).

### DMAb design and expression

The sequences of the single specificity anti-PcrV IgG (clone V2L2-MD) were obtained and optimized, as previously described (11). Previously, the nucleotide sequence for each human IgG1 heavy and Igκ light chains were codon optimized for both mouse and human to improve expression in mammalian cells. Sequences were also RNA optimized for improved mRNA stability translation efficiency by the ribosome (15, 33). To build off the previously reported single V2L2-MD plasmid, the optimized heavy and light chain genes were then inserted into separate pGX0001 (pVax) DNA expression vectors, under the control of a human cytomegalovirus promoter and bovine growth hormone polyA. It has been previously reported that this dual plasmid platform further improves expressed DMAb titers (28, 34, 35). Point mutations were made to the Wild-type Heavy Chain (HC) sequence to produce multiple complement modulated variants: (i) WT HC, (ii) E430G HC, and (iii) TM (L234F/L235E/P331S) HC. All HC plasmids were paired with the same V2L2-MD LC plasmid to produce fully formed and functional V2L2-MD DMAb variants.

HEK293T cells or Expi293F cells were transfected with DMAb DNA using either GeneJammer (Agilent, Cat# 204132) transfection reagent or Expi293 Transfection System (ThermoFisher Scientific; Cat# A14524), respectively. For HEK293T cell transfections, cell supernatants were harvested 48 hours post transfection. For Expi293F cell transfections, cell supernatants were harvested 96–120 hours post transfection. Samples were assayed for human IgG production by ELISA and Western Blot.

### Anti-Human IgG quantification ELISA

96-well, high-binding immunosorbent plates were coated with 5 µg/mL of goat anti-human IgG Fc antibody (Bethyl Laboratories, Cat# A80-104A) in 1X PBS and incubated overnight at 4C. The next day, plates were washed and blocked with 5% non-fat dry milk (NFDM) in 1X PBS for 1 hour at room temperature. Samples were serially diluted in ELISA diluent (1X PBS, 0.05% Tween 20, 1% Newborn Calf Serum (NCS)), plated in duplicate, and incubated at RT for 1 hour. Purified human IgG(λ) (Bethyl Laboratories; Cat# P80-112) was used to create a standard curve starting at 500 ng/mL diluted 2-fold. Bound antibodies were detected withanti-human IgG H+L antibody conjugated to horseradish peroxidase (HRP, Bethyl Laboratories; Cat# A80-319P) at 1:10,000 dilution and incubated for 1 hr at RT. Plates were developed with either o-Phenylenediamine dihydrochloride substrate (OPD, Sigma Aldrich,; Cat# P9187-50SET) or 1-Step Ultra TMB-ELISA (ThermoFisher Scientific; Cat# 34029), and stopped with 2N H_2_SO_4._ Plates were read at OD450 nm via BioTek Synergy2 plate reader (BioTek Instruments, Inc.). Plates were washed in between incubation steps with 1X PBS supplemented with 0.05% Tween 20 four times.

### Recombinant PcrV protein production

Recombinant PcrV protein was expressed and purified by Genscript Inc. (Piscataway, NJ) using a pET30a vector encoding PcrV-His. Protein was obtained from the supernatant of E. coli cell lysates and purified utilizing a Nickle column. The concentration was confirmed by Bradford assay. Quality control tests to assess purity (>90%, SDS-PAGE, reduced), size (SEC-HPLC), and intact mass (LC-MS) were also performed. Recombinant protein was shipped on dry ice and single use aliquots were stored at –80C.

### Recombinant PcrV binding ELISA

96-well, half-area, immunosorbent plates were coated with 2 µg/mL of recombinant *Pseudomonas aeruginosa* PcrV protein (Genscript, Piscataway, NJ) in 1X PBS and incubated overnight at 4C. The next day, plates were blocked with 5% NFDM in 1X PBS and incubated for 1 hour at room temperature. Purified V2L2-MD DMAb samples were used to assess binding to PcrV protein. All samples were started at 1 µg/mL and diluted 2-fold in duplicate. Samples were probed with anti-human IgG H+L antibody conjugated to HRP (Bethyl Laboratories; Cat# A80-319P) at 1:10,000 dilution and incubated for 1 hour at RT. Samples were developed with 1-Step Ultra TMB-ELISA and stopped with 2N H_2_SO_4._ Plates were read on the BioTek Syngery2 plate reader at OD450 nm. All plates were washed in between incubation steps with 1X PBS supplemented with 0.05% Tween 20 four times.

### Western blotting

Cell supernatants from DMAb-transfected cells were collected by centrifugation at 16,000 x g and transferred to new 1.5 mL Eppendorf tubes. Samples were quantified by anti-human IgG ELISA (see above) and 100 ng of DMAb supernatant was loaded on a 4-12% Bis-Tris SDS-PAGE gel (NuPage, ThermoFisher Scientific; Cat# NP0321). The gel was then transferred to a polyvinylidene difluoride (PVDF) membrane using the Invitrogen iBlot2 dry blotting system (ThermoFisher Scientific; Cat# IB24002). The membrane was then blocked with Li-Cor Intercept Blocking Buffer (PBS; Li-Cor; Cat# 927-70001) for 1 hr at RT shaking. V2L2-MD DMAbs were probed and detected with either IRDye CW800 Goat anti-human antibody (1:10,000) Li-cor; Cat# 926-32232) or Sigma donkey anti-human IgG h+l (Sigma Alrdich, 1:5000; Cat# SAB3701359) and incubated for 1 hr at RT shaking. Membranes were washed 3x for 15 minutes between incubations with 1X phosphate-buffered saline (PBS) with 0.01% Tween 20. Blots probed with Li-cor antibodies were imaged using the Odyssey CLX Imager (Li-Cor, Lincoln, NE). Blots probed with Sigma anti-human IgG h+l were treated with SuperSignal West Pico Plus chemiluminescent substrate (ThermoFisher Scientific; Cat# 34580) and imaged on the Amersham Imager 680 (General Electric).

### Gravity purification of expressed V2L2-MD DMAbs

Purified V2L2-MD DMAbs were prepared by gravity column purification using the rProtein A Sepharose Fast Flow resin per manufacturer’s protocol with minor modifications (Cytiva; Cat# 17127902). Expi293F transfection supernatants were incubated with resin overnight at 4°C, rotating. The next day, supernatants were filtered using gravity flow columns (Bio-Rad; Cat# 7321010). Resin was then washed with 1X PBS pH8 at 5x-10x the concentration of resin volume. Labeled tubes were prepared with 1M Tris HCl pH8 in preparation for the elution step. To remove bound V2L2-MD DMAb, resin was washed with Elution Buffer (20mM Citrate). IgG1 concentration was from eluate was determined using the IMPLEN NanoPhotometer NP80 (IMPLEN). Purified supernatants were then buffer exchanged in 1X PBS pH 7.4 using Amicon Ultra Centrifugal Filters (50K, Millipore Sigma; Cat# UFC905008). Used resin was regenerated with 1M Glycine-HCl pH2–3 and stored at 4°C in 20% Ethanol. Flow through from each step after incubation of resin with supernatant was saved and concentration of IgG1 was verified by nanophotometer.

### Complement binding C1q ELISA

To test binding of our V2L2-MD DMAbs to complement protein, clear 96-well flat-bottom half area plates (Corning) were coated with recombinant Native Human C1q protein (Creative BioLabs; Cat# CTP-461at a concentration of 50 µg/mLin 1X PBS and incubated overnight at 4C. The next day, wells were blocked with 5% NFDM-1X PBS and incubated at room temperature fo1 hr. Purified DMAb samples were prepared in diluent at 500 µg/mL and diluted 2-fold. Samples were incubated at room temperature for 1 hour. Bound antibodies were detected with anti-human IgG h+l antibody conjugated with HRP (Bethyl Laboratories) diluted 1:10,000 at RT for 1 hour. Plates were developed using 1-Step Ultra TMB-ELISA (ThermoFisher Scientific) and stopped with 2 N H_2_SO_4._ Plates were read using a BioTek Synergy 2 plate reader (BioTek Instruments Inc.) at 450-nm wavelength and binding curves were assessed. All wells were washed with washed with 1× PBS–0.05% Tween 20 between incubations and all samples were serially diluted in 1× PBS–0.05% Tween–1% Newborn Calf Serum (NCS) diluent.

### Pseudomonas aeruginosa strain PAO1 and PA14 culturing for killing assays

*Pseudomonas aeruginosa* strain PA14 and PAO1 broth cultures were grown at 37°C, shaking in Lysogeny Broth (LB) (Miller’s formulation; Corning; Cat# 46-050-CM) overnight. 25% glycerol stocks were made in 1 mL aliquots and frozen at -80C until used. Glycerol stocks were used to streak LB agar plates which were incubated overnight at 37°C. The next day, single colonies were picked and grown in 4 mL of LB Broth shaking at 37°C for 4 hours. 4-hour broth cultures were diluted and read at OD600 to determine absorbance. Dilutions with an OD600 0.3-0.075 were serially diluted 10-fold. 10-fold dilutions per each absorbance were plated on LB agar plates and incubated overnight. The numbers of CFU per milliliter of these OD600 dilutions were determined by the number of colonies on the LB agar plates the next day. These CFU/mL values were used to determine the number of bacteria to add to the opsonophagocytic killing assays.

### ADCP, ADCD, and cADCP killing assay

The bacterial killing assay was performed as described with minor modifications (36–38). PA14 or PAO1 was incubated in triplicate wells in a 96-well round bottom TC treated plate for 1 hr at 37°C with purified V2L2-MD DMAbs (200 µg). Human promyelocytic leukemia cell line HL-60 (ATCC) were cultured in IMDM (ATCC) with 20% heat-inactivated FBS (PeakSerum). HL-60 cells were differentiated with 0.6% *N,N*-dimethylformamide (DMF Sigma-Aldrich; Cat# 227056) for 3–4 days. Differentiation of HL-60s was confirmed via flow cytometry, as described (36). Cells were stained with PE-CD35 (Biolegend; Cat# 333406) and APC-CD71 (Biolegend; Cat# 334108) and analyzed by flow cytometry. Differentiation was determined successful with a cell population >65% CD35^+^ and <20% CD71^+^. The day of the assay, colonies were picked and grown in 4 mL broth culture, shaking, for 4 hours at 37C. After incubation, bacteria were diluted to an OD600 of 0.075-0.085 and further diluted 10–^4^ to achieve an optimal number of colonies per plate (approximately 150–300 CFUs/50 µL). Differentiated HL-60 cells were harvested and resuspended in 1 mL opsonization buffer B (OBB; sterile 1x PBS + Ca^2+^/Mg^2+^, 0.1% sterile gelatin, 5% heat inactivated FBS). Cells were further diluted 1:4 in OBB and this diluted cell suspension was used for the assay. Baby Rabbit complement (Pel-Freeze; Cat# 31065) was diluted in OBB at dilution of 1:50. All components of the killing assay (HL-60 cells, WU2, baby rabbit sera, and antibody) were added at final volumes of 25 µL in OBB. No antibody control plates contained only the diluted HL-60 cells, rabbit sera, and PA14 with 25 µL of non-antibody OBB for a final volume of 100 µL. The final reaction mixtures were incubated at 37°C for 1 hour. 50 µL of each reaction mixture (in triplicate) were plated on LB agar plates and incubated overnight at 37C and CFU per plate were counted the next day. For the ADCD killing assay, no differentiated HL-60 cells were added, and the final volume was brought up to 100 µL/well with OBB. For the ADCP killing assay, no complement was added, and the final volume was brought upt to 100 µL/well with OBB. Percent bacterial killing was calculated as the value for each replicated normalized to the mean of the no antibody-control wells ((Normalized Mean Control - # colonies in Ab mixture triplicate)/Normalized Mean Control) *100. No antibody control samples represent 0% bacterial killing.

### Antibody-mediated complement deposition assay

To evaluate the ability of V2L2-MD DMAbs to induce deposition of downstream C3 complement protein, recombinant PcrV protein was biotinylated with EZ-Link™ Sulfo NHS-LC-LC-Biotin (ThermoFisher Scientific; Cat# A35358) and coupled to 1.0µm FluoSpheres NeutrAvidin beads (ThermoFisher Scientific; Cat# F8775). The antigen-coupled beads were incubated with 1X PBS diluted DMAb (5 µg/ml, 1 µg/ml, 0.2 µg/ml) or RPMI1640 (Corning) diluted mouse serum samples (1µg/ml) at 37°C for 2 hours. Unbound antibodies were removed by centrifugation after incubation. An aliquot was taken out and heat-inactivated at 56°C for 30 min after low-tox-guinea pig complement (Cedarlane; Cat# CL4051was reconstituted. Both active and heat-inactivated aliquots were diluted in gelatin veronal buffer with Ca^2+^ and Mg^2+^ (Complement Technology; Cat# B100) and incubated with the beads for 20 min at 37°C. Beads were washed with 1X PBS +15mM EDTA for three times and stained with FITC-goat anti-Guinea Pig C3 antibody (MPBio; Cat# 0855385C3 deposition on beads was analyzed by a Cytek Aurora Spectral Flow Cytometer. The Median Fluorescence Intensity (MFI) of C3 on beads was measured. Cytek SpectroFlo software was used for the data analysis. The heat-inactivated MFI was subtracted from the active MFI to account for non-specific deposition.

### Acute murine PAO1 challenge model

Female BALB/c mice, aged 6–8 weeks (n=10-20) were purchased from Charles River Laboratories and administered V2L2-MD DMAbs in doses by IM-EP (12.5 µg, 25 µg, 50 µg, 100 µg) or anti-Ebola DMAb-11 (25 µg or 50 µg) control on D0. A parallel group of mice per group (n=5-10) were kept and administered matching challenge doses and bled to verify expression by ELISA on D28 pre-shipment for challenge. *P.* strain PAO1 was grown from a frozen stock on lysogeny agar (LA, Miller formulation) at 37°C overnight. A 3ml LB tube was then inoculated with a single colony and incubated at 37°C overnight, before being diluted 1:100 into 3mL fresh LB and incubated at 37°C for 4 hours to achieve exponential growth. Following exponential growth, bacteria were resuspended in 1x phosphate-buffered saline (1X PBS) prior to challenge. Prior to challenge, mice were anesthetized with 200µL of ketamine (Patterson Veterinary; #07-803-6637, ~77mg/kg of body mass) and xylazine (Bayer; #047-956, ~7.7mg/kg of body mass) in 0.9% saline via intraperitoneal injection. On D35-D42 post-DMAb administration, animals received 20µL of the bacterial suspension containing 5x10^6^ CFUs of *P. aeruginosa* PAO1. Approximately 14–16 hours post-challenge, mouse body temperatures were collected via rectal temperature probe and mice were euthanized with 200µL of Euthasol^®^ Euthanasia solution (Patterson Veterinary; #07-805-9296, ~390mg pentobarbital/kg of body mass) in 0.9% saline via intraperitoneal injection. Following euthanasia, blood was collected via cardiac puncture, lungs were aseptically removed and weighed, and nasal wash was collected by flushing 1mL of sterile 1X PBS through the nasal cavity. Lungs were homogenized using a gentleMACS™ Tissue Dissociator (Miltenyi Biotec). Lung homogenate and nasal wash were then serial diluted and plated onto Pseudomonas Isolation Agar (PIA), then grown at 37°C overnight before CFU enumeration.

### Software and statistical analysis

Data was represented in GraphPad Prism version 10. Details on statistical analysis are included in the legend for each figure.

## Results

### Two plasmid delivery of Fc-WT V2L2-MDDMAb

**Figure 1:**
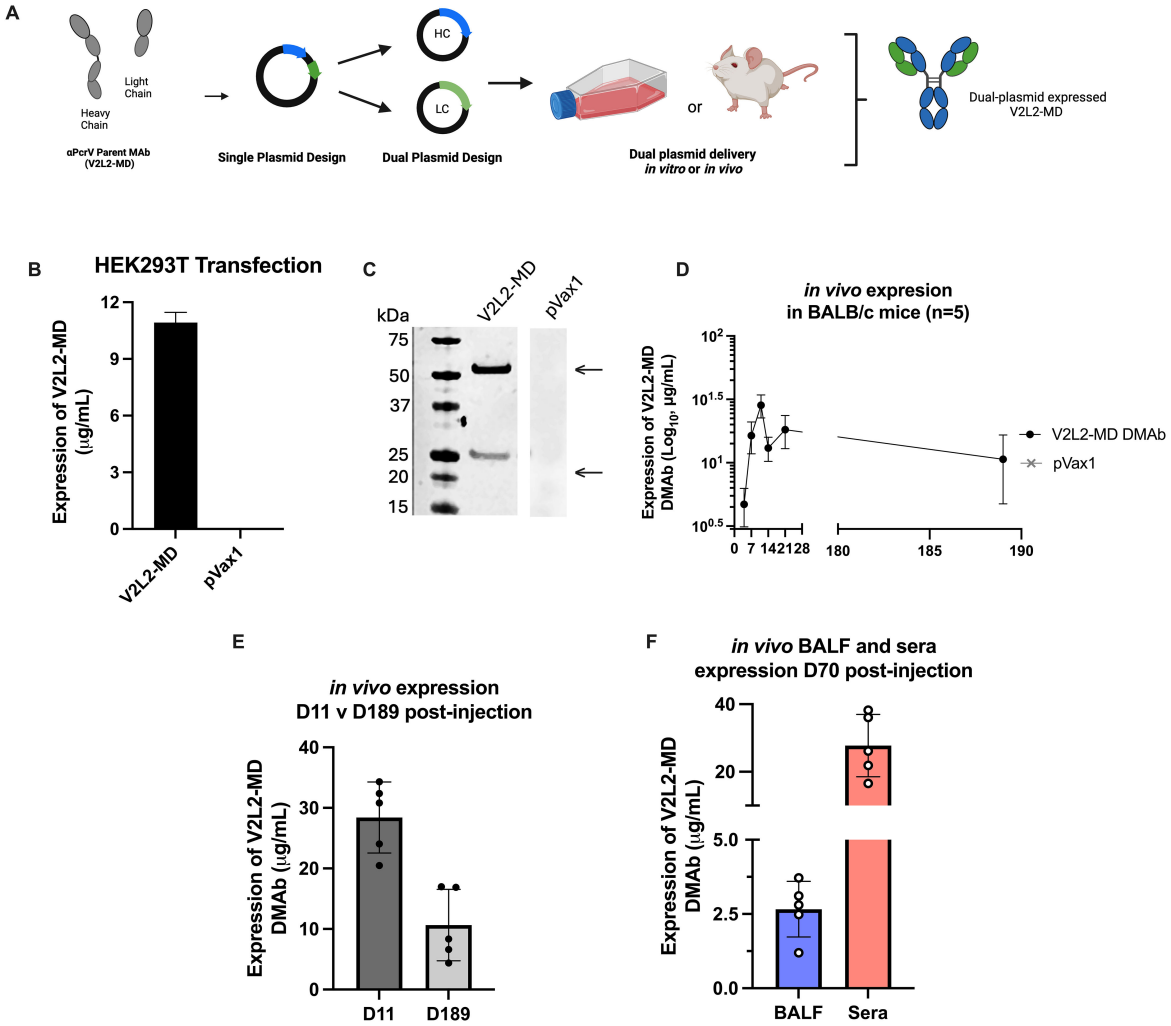
Design and characterization of wild type Fc (Fc-WT) V2L2 DMAb on dual plasmid system. **(A)** Diagram of Fc-WT V2L2 DMAb encoding the hIgG1 heavy chain (HC) and light chains (LC) a single plasmid or two separate plasmids **(B)** Fc-WT V2L2 DMAb or empty vector pVax1 expression in transfected HEK293T cells. **(C)** Western blot of supernatants from transfected cells run under reducing conditions. Bands indicating V2L2 HC and LC are detected at 55 kDa and 25 kDa, respectively. V2L2-MD DMAb and pVax1 samples were run on the same blot, but not side by side. **(D)** ELISA demonstrating long-term expression of Fc-WT V2L2 DMAb in mouse sera (n=5/group). **(E)** *in vivo* expression of WT V2L2 DMAb at D11 (peak expression) versus D189. **(F)** *in vivo* expression of WT V2L2 DMAb at D70 in bronchioalveolar lavage fluid (BALF) and sera quantified by ELISA (n=5).

Previously, we described re-engineering of MAb V2L2-MD as the synthetic DMAb-αPcrV encoding both heavy (HC) and light (LC) chains into a single plasmid DNA expression vector with additional nucleotide and RNA sequence optimizations for *in vivo* gene-encoded MAb delivery (11, 15). To facilitate study of Fc-modified variants, we separately encoded the human immunoglobulin gamma 1 (hIgG1) HC and LC genes of parent V2L2-MD DMAb (Fc-WT V2L2-MD DMAb) onto separate plasmids, first confirming successful DMAb expression (**Figures 1A–C**). Long-term *in vivo* expression of dual plasmid delivery V2L2-MDMAb was assessed by administering V2L2-MD DMAb DNA (25 µg HC + 25 µg LC) to BALB/c mice by intramuscular injection followed by CELLECTRA-3P electroporation (IM-EP), with *in vivo* expression achieving peak serum levels of human IgG exceeding 20 µg/mL by day 11. V2L2-MD DMAb was detectable six-months post-administration with 80% of animals expressing >6 µg/mL (**Figures 1D, E**). V2L2-MD DMAb was also detected in bronchioalveolar lavage fluid (BALF) collected D70 post-administration, demonstrating hIgG1 levels in all animals, indicating that *in vivo*-expressed DMAbs circulate through the blood to the site of infection prior to challenge. Of these mice, 4/5 mice expressed V2L2-MD DMAb >2 µg/mL in BALF. All mice had detectable levels of V2L2-MD DMAb >15 µg/mL in sera at D70 (**Figure 1F**). Together these data show successful *in vivo* production of V2L2-MD DMAb on a dual plasmid system with expressed DMAb detectable in the lungs.

### Delivery of Fc-WT V2L2-MD DMAb significantly decreases bacterial load in an acute PAO1 murine challenge model

**Figure 2:**
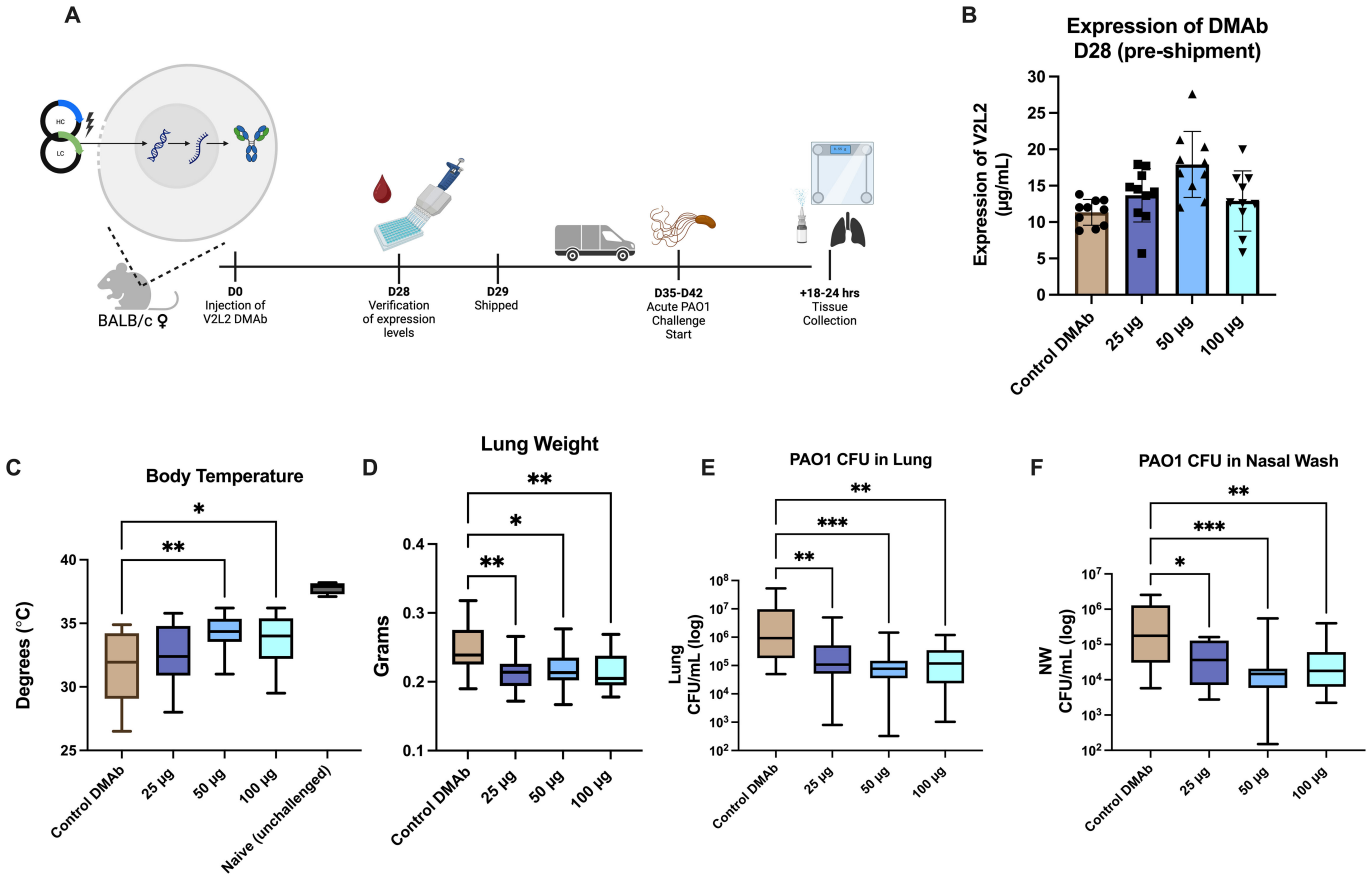
Fc-WT V2L2-MD DMAb in doses in an acute PAO1 challenge model. **(A)** Overview of the PAO1 challenge timeline. Mice were administered Fc-WT V2L2-MD DMAb at a dose of 25 µg, 50 µg, or 100 µg (n=10/group) on D0. After 28-days, mice were bled (n=9-10/group) for verification of expression of DMAb by quantification ELISA. Mice were then shipped to WVU for challenge on D35-D42 post-injection. **(B)** Quantification of *in vivo* expressed Fc-WT V2L2-MD DMAb on D28-post injection. Control mice were delivered an Ebola-targeting DMAb-11 at a 50 µg dose. **(C-F)** Body temperature, lung weight, CFUs/lung, and CFU/nasal wash were assessed 18-24 hours post challenge in groups delivered Fc-WT V2L2-MD DMAb compared to the control DMAb group. Statistical analysis performed by Kruskal-Wallis with Dunnett’s multiple comparison test (* *p<0.05*, ** *p<0.01*, *** *p<0.001*). Data are representative of two independent experiments.

Previously, we evaluated the functional activity of single plasmid DNA-delivered V2L2-MD DMAb against pathogenic and cytotoxic *P. aeruginosa* strain 6077 using a lethal murine pneumonia model demonstrating V2L2-MD protection and decreased bacterial burden in multiple organs (15). However, these challenges did not test functionality of V2L2-MD DMAb in an acute infection setting where the Type III Secretion System (T3SS) and PcrV are upregulated. During acute infections, both have been correlated to poor patient outcomes, increased bacterial burden, and bacterial persistence (12, 39). To address this question, we performed a lethal, acute intranasal challenge with Fc-WT V2L2-MD DMAb and the *P. aeruginosa* PAO1 strain in mice (**Figure 2A**). Mice received Fc-WT V2L2-MD DMAb at a 25 µg, 50 µg, or 100 µg dose or negative control anti-Ebolavirus glycoprotein DMAb-11 at 50 µg (34). At D28 post-administration, animals were bled to confirm human IgG1 DMAb titers and then shipped to West Virginia University for challenge (**Figure 2A**). Prior to shipping, mouse expression of Fc-WT V2L2-MD DMAb was detected in a dose-dependent manner (**Figure 2B**). Animals were administered 5 x 10^5^ CFU of PAO1 intranasally and euthanized at 14–16 hours post-infection.

Body temperature was monitored at the end of challenge as mice typically exhibit hypothermia, lowered body temperatures, as a survival strategy to combat infection, particularly sepsis, in a bacterial infection setting (40–43). Healthy unchallenged mice had an average body temperature around 37°C; meanwhile, negative control Ebola DMAb-11 administered mice showed a significant decrease in body temperature (32°C, *p<0.0001*). Mice administered Fc-WT V2L2-MD DMAbs, specifically 50 µg or 100 µg, had significantly higher body temperatures compared to the control group (**Figure 2C**, *50 µg p=0.0079, 100 µg p=0.0406*). Upon necropsy, we assessed lung weight which often increases during infection due to edema and the inflammatory response (25, 44). Mice delivered Fc-WT V2L2-MD DMAbs at all doses showed significantly lower lung weights, in a dose-dependent manner, compared to the control DMAb group (**Figure 2D**, *p<0.02)*. Animals administered all doses of Fc-WT V2L2-MD DMAb showed a significant decrease in overall bacterial burden in both the lung and nasal washes compared to animals administered the control DMAb (**Figures 2E, F**, *p<0.007* and *p<0.036*, respectively). Overall, mice administered 50 µg and 100 µg of WT V2L2-MD DMAb had approximately a 10-fold decrease overall bacterial lung burden compared to the control. All V2L2-MD DMAb groups, regardless of dose, had approximately a 10-fold decrease in bacterial burden in the nasal wash compared to the control mice. Together, these data show the Fc-WT V2L2-MD DMAb delivered at all doses can improve acute PAO1 challenge outcomes.

### Design and expression of complement-modulated V2L2-MD DMAb Fc variants

**Figure 3:**
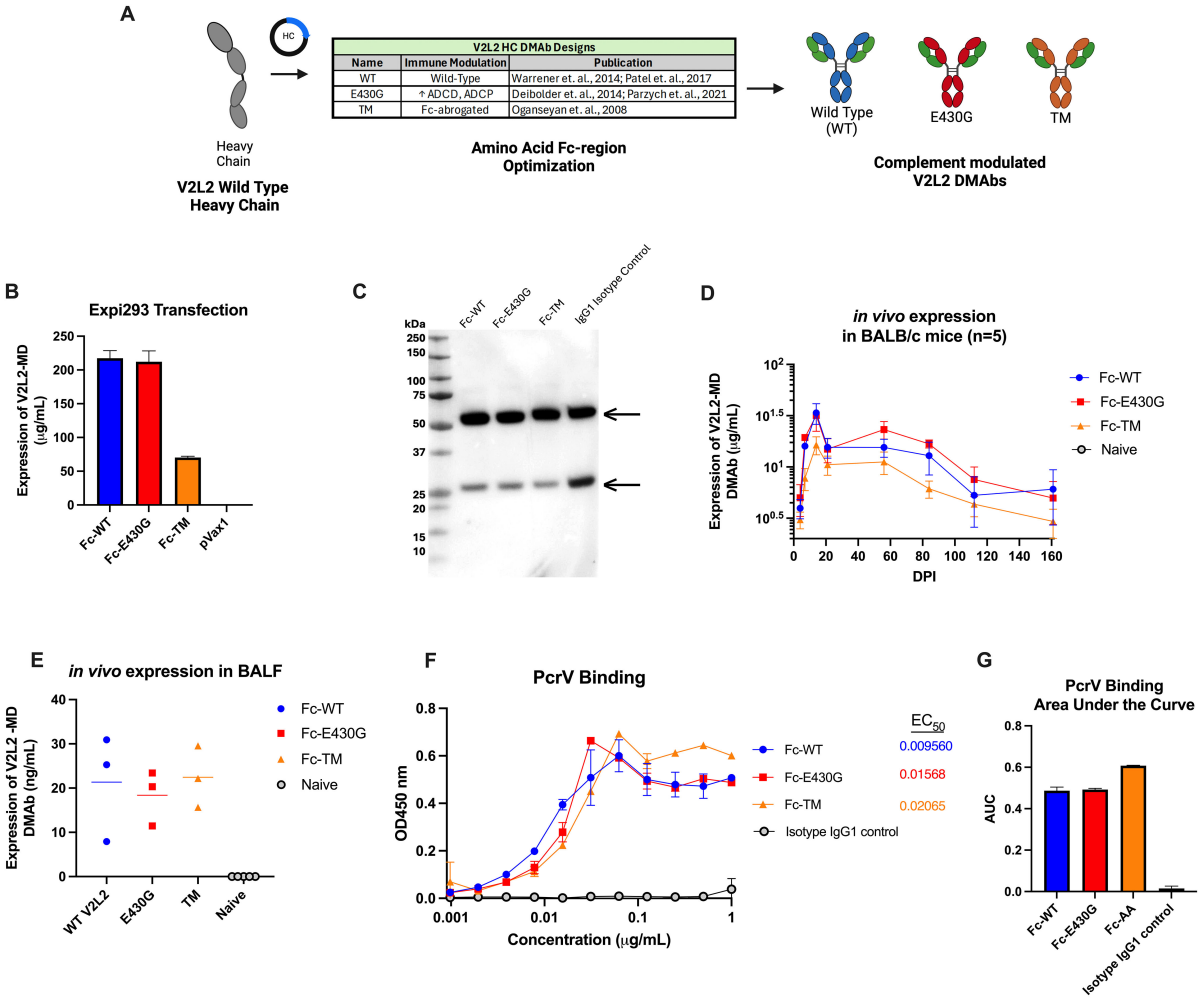
Design and expression of complement modulated V2L2 DMAbs. **(A)** Graphical schematic showing the V2L2 wild-type heavy chain sequence and plasmid design to generate complement modulated variants of V2L2-MD by inserting point mutations that will either increase or decrease C1q binding. **(B)** Expi293T cells were transfected with 1 µg DNA per 1 mL of transfection volume with designed V2L2 DMAb plasmids. Supernatants were harvested 5-days post transfection start. Expression of V2L2 mAb variants was assessed via quantification ELISA (group mean ± SD) **(C)** Western blot was performed with supernatant from transfected cells run on an SDS-PAGE gel under reduced conditions and transferred to a PVDF membrane. The blot was probed with goat anti-human IgG (Licor) and visualized via OdysseyCLX Imager. **(D)** Mice (n=5/group) were administered 50 µg of V2L2 DMAb or empty vector pVax control by IM-EP. Mice were bled days post injection (DPI) and expression was assessed via quantification ELISA of mouse sera (group mean ± SD). **(E)** Mice (n=5/group) were administered 50 µg of V2L2 DMAb and on D21 DPI bronchioalveolar lavage fluid was taken and expression of V2L2 DMAbs was assessed via quantification ELISA compared to Naïve mice. **(F)** Purified V2L2 DMAbs were used to assess binding to PcrV protein (coated 5 µg/mL). Calculated EC50 values are displayed. All samples started at 1 µg/mL (diluted 2-fold; group mean ± SD). **(G)** Calculated Area Under the Curve (AUC) of PcrV binding for all V2L2 DMAb variants. All data analysis performed using GraphPad Prism 10.

Fc-point mutations have been shown to alter effector functions of antibodies including complement-mediated clearance. The parent V2L2-MD DMAb HC sequence was modified to incorporate sequence changes hypothesized to enhance or abrogate complement activation (**Figure 3A**). The first V2L2-MD HC variant included a single amino acid change of a glutamic acid (E) at position 430 to a glycine (G) (E430G), in the Fc CH_2_-CH_3_ interface, that has been shown to improve ADCD and ADCP (red) (27, 28, 45). The second variant, TM V2L2-MD (orange), contains three mutations in the CH_2_ region (L234F/L235E/P331S) to generate a fully Fc-silenced version incapable of immune receptor or complement binding (46). All HC-variants were paired with the parent V2L2-MD DMAb LC.

Expression of antibody from co-delivered HC-variant and LC plasmids was confirmed by Expi293F cell transfection, followed by detection via an anti-human IgG ELISA and Western Blot utilizing the harvested supernatants. All V2L2-MD DMAb variants expressed similarly, except for the TM variant which displayed lower expression. The respective IgG1 heavy chains (55 kDa) and light chains (25 kDa) for all Fc-variants and IgG1 isotype control were detected by western blot under reduced conditions, suggesting proper expression and secretion of DNA-encoded V2L2-MD MAb variants (**Figures 3B, C**).

Next, we evaluated the pharmacokinetics of V2L2-MD DMAb variants administered in BALB/c mice. Mice (n=5/group) were administered a total DNA dose of 50 µg of V2L2-MD DMAb followed by serum concentration monitoring expressed DMAb over time post-single injection. Peak expression levels observed for all animals at D14, with expression >6-months post-administration. Mice administered the Fc-WT or Fc-E430G variants exhibited peak expression between 20-45 µg/mL; however, animals delivered V2L2-MD variant containing the TM modification showed lower DMAb expression (**Figure 3D**). In a parallel cohort of DMAb administered mice (n=5/group), BALF was taken on D21 post-administration to determine the levels of expressed DMAb in the lungs, confirming the presence of expressed V2L2-MD variants at the site of infection (**Figure 3E**). To confirm that the addition of Fc-modifications did not disrupt antigen binding, V2L2-MD DMAb variants were assessed for binding to recombinant PcrV protein. All DMAbs exhibited similar binding profiles, with comparable EC50 values and area under the curve (AUC) measurements (**Figures 3F, G**).

Overall, *in vitro* assays confirm successful expression and secretion of Fc-modified V2L2-MD DMAbs that are capable of binding target PcrV protein.

### *In vitro* assays show that Fc-E430G improves complement activation mediated by the V2L2-MD DMAb

**Figure 4:**
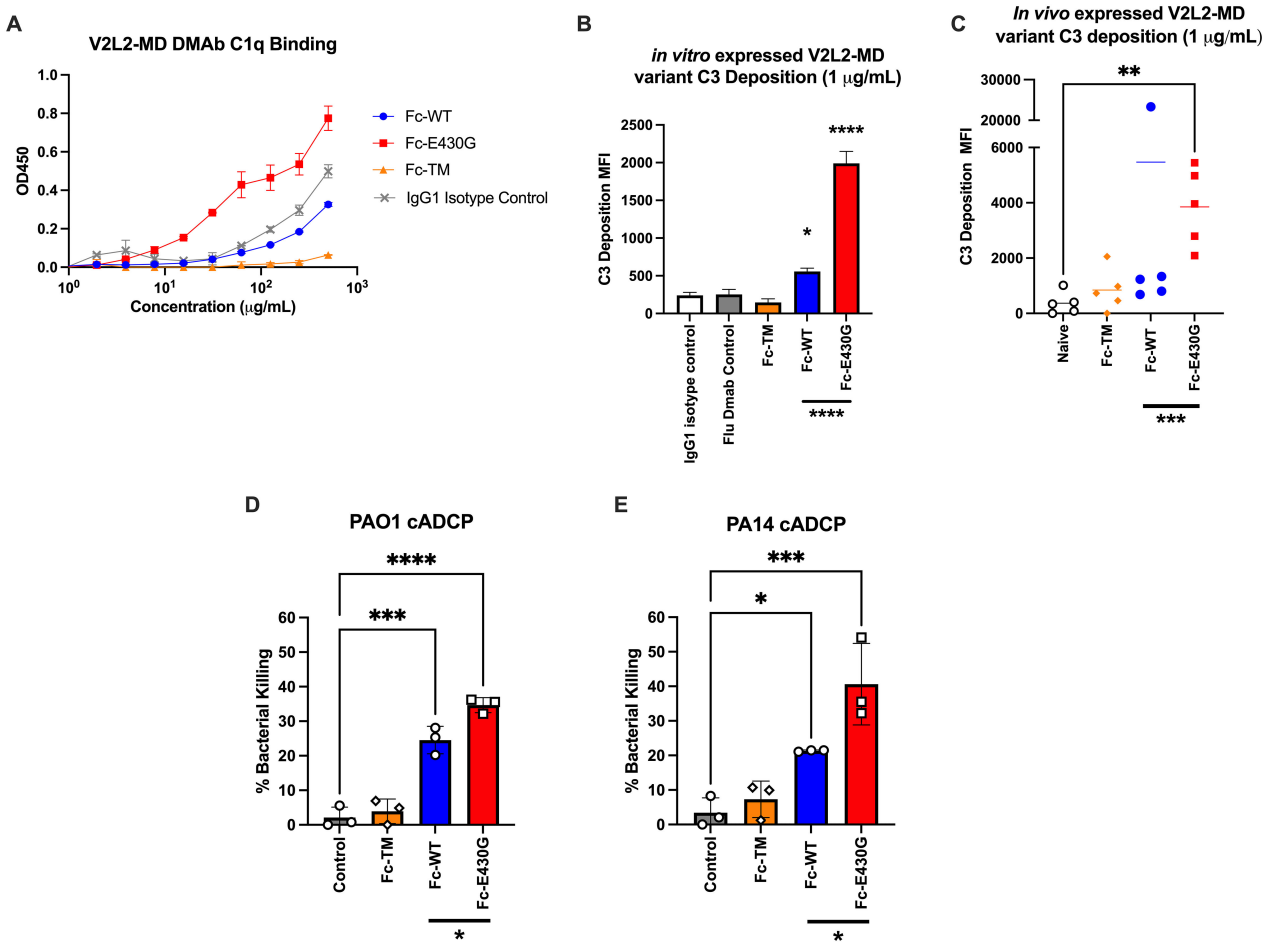
Fc-engagement with complement proteins and assessment of DMAb functionality. **(A)** V2L2-MD DMAb variant binding to C1q (50 µg/mL) coated ELISA plates. Purified V2L2-MD DMAbs were prepared at a concentration of 0.5 mg/mL (diluted 2-fold; group mean ± SD). **(B)** Purified V2L2-MD DMAb Variant Fc-binding guinea pig complement and probed with FITC-conjugated anti-guinea pig C3 antibody compared to Fc-abrogated TM V2L2, Flu DMAb 2-12C control, and IgG1 Isotype Control (n=2; group mean ± SD). **(C)** *in vivo* produced V2L2-MD DMAb C3 deposition was measured utilizing guinea pig complement and probed with a FITC-conjugated anti-guinea pig C3 antibody. All variants were compared to sera from naïve mice (n=5/group; group mean ± SD). Outliers identified (>20,000 MFI, blue) in GraphPad Prism 10 (Q = 1%). Statistical analysis performed on outlier excluded data. **(D)** PAO1 and **(E)** PA14 complement-mediated antibody-dependent cellular phagocytic (cADCP) killing assay utilizing purified V2L2 DMAb variants at 200 µg dose. Percent (%) bacterial killing was calculated as ((mean of control - # DMAB variant colonies)/mean control)*100 (n=3/group). Negative percent bacterial killing represents bacterial growth. Statistical analysis performed in GraphPad Prism 10 by one-way ANOVA (* *p<0.05*, ** *p<0.01*, *** *p<0.001*, **** *p<0.0001*).

The classical complement pathway is initiated by the Fc-binding of C1 multimeric protein (C1q, C1r_2_, C1s_2_) to antibody-antigen complexes resulting in downstream phagocytosis or MAC formation (47). Although wild-type V2L2-MD DMAb is capable of conferring protection during *P. aeruginosa* infection, we hypothesized that the complement enhancing Fc-mutation E430G can further improve bacterial clearance V2L2-MD DMAb via induction of complement-mediated antibody-dependent cellular phagocytosis (cADCP) or antibody-dependent complement deposition (ADCD). We first assessed the binding of purified monomeric V2L2-MD DMAb variants to native human C1q protein compared to IgG1 isotype control. Fc-abrogated (TM) V2L2-MD DMAb exhibited a loss of binding to C1q protein while the Fc-WT V2L2-MD, Fc-E430G V2L2-MD, and isotype control all show binding. Overall, Fc-E430G V2L2-MD DMAb exhibited the highest binding for C1q protein (**Figure 4A**). To further assess downstream complement pathway activation, an ADCD assay was performed to analyze V2L2-MD DMAb C3 deposition using PcrV-coated beads. Purified DMAbs (1 µg/mL) were combined with PcrV coated beads and guinea pig complement, followed by probing with a FITC anti-guinea pig C3 antibody. C3 deposition was measured via median fluorescence intensity (MFI). For *in vitro* produced DMAb, minimal C3 deposition was observed by Fc-TM V2L2-MD DMAb, Isotype control, and Flu DMAb control. Similarly to the C1q binding ELISA, C3 deposition was observed by the Fc-WT and E430G V2L2-MD DMAbs; however, Fc-E430G V2L2-MD DMAb exhibited a significant difference in C3 MFI compared to Fc-TM and WT, respectively (0.2 to 5 µg/mL; *TM p<0.0001, WT p<0.0001;* **Figure 4B**). C3 deposition of *in vivo* produced V2L2-MD DMAbs demonstrated a significant increase in C3 deposition by Fc-E430G V2L2-MD DMAb compared to Fc-WT V2L2-MD, or Fc-TM V2L2-MD, and naïve sera samples (**Figure 4C**, *WT p=0.0006, TM p=0.0002, Naive p<0.0001*).

Having demonstrated that Fc-modification E430G can improve complement binding, we south to determine whether this translates to increased bacterial killing. Here we utilized our challenge model strain PAO1 and common laboratory strain PA14, as both are frequently used to assess novel therapeutics (48). First, purified V2L2-MD DMAbs were incubated with either PA14 or PAO1, differentiated HL-60 cells, and baby rabbit sera as a complement source to measure complement-mediated antibody dependent cellular phagocytic killing (cADCP; **Figures 4D, E**). As anticipated, the Fc-abrogated TM variant exhibited no bacterial killing against either *P. aeruginosa* strain. Meanwhile, a significant increase in bacterial killing was observed by both the Fc-WT and Fc-E430G DMAbs when compared to no antibody control (**WT,** PAO1 *p=0.001*, PA14 *p=0.0304;* **E430G**, PAO1 *p<0.0001*, PA14 *p=0.0004)*. Notably, the Fc-E430G DMAb variant also demonstrated superior killing relative to Fc-WT DMAb for both *P. aeruginosa* strains (PAO1 *p=0.0216;* PA14 *p=0.0345*).

To further investigate bacterial killing by other Fc-dependent mechanisms, additional *in vitro* killing assays were performed to assess antibody-dependent cellular phagocytosis (ADCP; no complement) and antibody-dependent complement-deposition (ADCD; no phagocytes) (**Supplementary Figures S1A–D**). In the ADCP assays, no significant difference in bacterial killing was observed between DMAb-treated and no antibody control groups, suggesting that Fcγ receptor-mediated phagocytosis alone does not contribute to bacterial killing in this setting. Furthermore, in the ADCD killing assays, both the Fc-WT and Fc-E430G DMAbs perform significant bacterial killing relative to control (**WT,** PAO1 p=ns, *0.0644*, PA14 p=*0.0160*; **E430G**, PAO1 *p=0.0124*, PA14 *p=0.0143*). However, no significant difference in killing was observed between the Fc-WT and Fc-E430G variants, indicating that while membrane attack complex (MAC)-mediated killing occurs via complement activation, the superior activity of Fc-E430G DMAb is displayed in the cADCP assays.

Together these data show that the inclusion of the E430G Fc-modification enhances V2L2-MD DMAb complement binding, leading to improved complement-mediated phagocytic killing of *P. aeruginosa*.

### Delivery of the complement modulating Fc-E430G V2L2-MD DMAb improves antibody activity in an acute PAO1 challenge model

**Figure 5:**
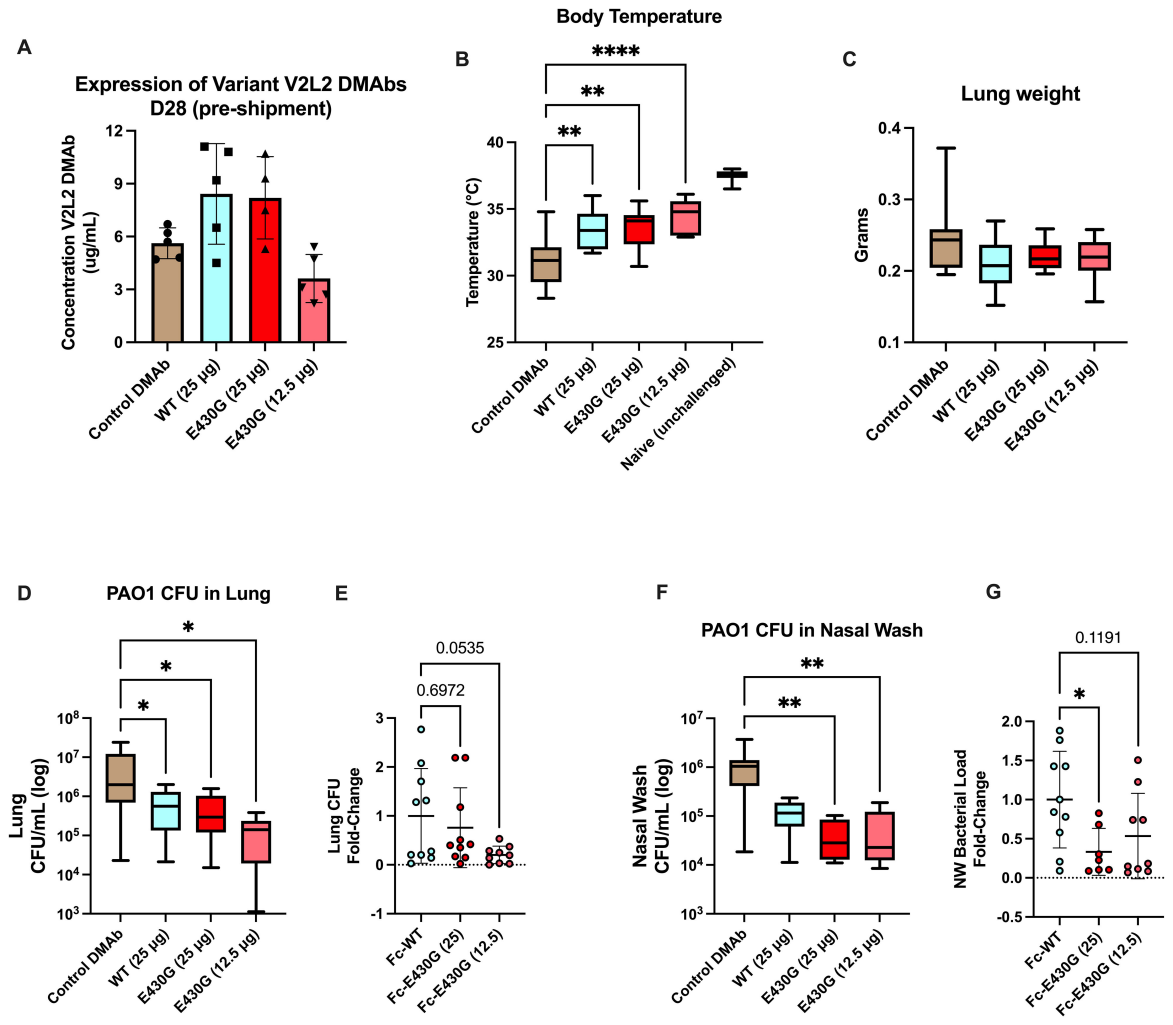
Low dose delivery of complement modulated Fc-E430G V2L2-MD DMAb variants in doses in an acute PAO1 challenge model. Mice were administered either Fc-WT or E430G DMAb at one of two lower doses (25 µg, 12.5 µg), respectively. Control mice were administered Ebola DMAb-11 at a 25µg dose. **(A–C)** Serum concentration (n=4-5), body temperature, and lung weight of BALB/c intranasally challenged with PAO1. **(D)** Bacterial load in the lung of challenged mice. **E)** Fold-change in lung bacterial load of Fc-WT and Fc-E430G DMAb mice. **(F)** Bacterial load in the nasal wash of challenged mice. **(G)** Fold-change in nasal wash bacterial load of Fc-WT and Fc-E430G DMAb mice. Outliers were identified using GraphPad Prism 10 (Q = 1%). Statistical analysis was performed after outlier identification via one-way ANOVA or Kruskal-Wallis with Dunnett’s multiple comparison test (* *p<0.05*, ** *p<0.01*, **** *p<0.0001*).

Using the PAO1 challenge model we administered Fc-E430G or Fc-WT V2L2-MD DMAb WT V2L2-MD DMAb at 12.5 µg or 25 µg, or control anti-Ebola DMAb-11 (n=10/group). All animals display expression of DMAb in mouse sera prior to shipment for challenge (**Figure 5A**). Unchallenged mice exhibited an average body temperature of 37°C. A significant improvement in body temperature was detected in all animals delivered V2L2-MD DMAb compared to control Ebola DMAb-11 (32°C, *WT 12.5 µg p=0.0029, E430G 12.5 µg p<0.0001, E430G 25 µg p=0.0017*). All animals delivered V2L2-MD DMAbs had a significant increase in overall body temperature compared to the control animals with the highest average body temperature in the 12.5 µg Fc-E430G V2L2-MD DMAb group (**Figure 5B**, *p<0.0001*). Furthermore, all animals delivered V2L2-MD DMAbs show trending decreases in lung weight compared to control (**Figure 5C**). Overall bacterial burden in the lung and nasal wash was significantly decreased in animals administered V2L2-MD DMAbs. Animals expressing Fc-E430G V2L2-MD DMAb had the largest reduction in bacterial load compared to Fc-WT and control groups (**Figures 5D, F**). Here mice administered 12.5 µg and 25 µg of Fc-E430G DMAb exhibited the largest reduction in bacterial load in the lung and nasal wash, respectively, (~1.5 log reduction; p=0.0198; p=0.0012) when compared to control; meanwhile, animals delivered Fc-WT V2L2-MD DMAb exhibited ~1-log decrease in overall bacterial burden compared to control. In comparison to the parental Fc-WT DMAb, majority of animals delivered either dose (25 µg or 12.5 µg) of Fc-E430G DMAb exhibit bacterial burden less than the average CFU/mL observed in the Fc-WT group in the lung and nasal wash. More importantly, a marginally significant reduction in fold-change of lung CFUs was observed in the 12.5 µg Fc-E430G DMAb (*p=0.0535*) when compared to Fc-WT DMAb with a significant reduction in the nasal wash in animals given 25 µg Fc-E430G (*p=0.0316*) compared to WT-Fc (**Figures 5E, G**; **Supplementary Figures S2** and **S3**).

Overall, prophylactic delivery of V2L2-MD DMAbs can improve acute PAO1 challenge outcomes, with the E430G-Fc modification further reducing overall bacterial load.

## Discussion

Antimicrobial resistance continues to be one of the largest public health threats with the rapid global spread of “superbugs” with high resistance profiles to an array of antibiotics (49). Despite their importance in combating bacterial infections, preemptive use of antibiotics, along with challenges to access of appropriate medicines during the SARS-CoV-2 pandemic, has contributed to an unanticipated rise in antimicrobial resistance profiles for multiple pathogens (50, 51). Ultimately, this extraordinary global event has impacted resistance profiles amongst multiple bacterial pathogens (5). A large proportion of these pathogens are commonly treated with broad-spectrum antibiotics where inappropriate use can lead to the development of new resistant strains (52, 53). Furthermore, the World Health Organization previously published an analysis report summarizing pipeline findings and recommendations for the development of vaccines against AMR pathogens (54). Many of these pathogens have low feasibility for vaccine development mainly due to biological complexities, difficulty to recruit critically ill target populations, and no current precedent for routine vaccinations of patients in intensive care. For example, while many anti-*P. aeruginosa* vaccine antigens are variable in protection, historically many are associated with adverse events or high antibody titers correlated with poorer clinical outcomes, especially in high-risk Cystic Fibrosis patients (55–60). Therefore, many of these pathogens, including *P. aeruginosa*, fall under the Group D category where it is suggested alternative interventions be explored.

With a growing need for medical interventions to combat these infections, development of protective MAbs has emerged as a promising alternative AMR strategy that has been FDA approved for protection in high-risk, immunocompromised patients. Generally, recombinant MAbs are administered and circulate to offer protection against a future or ongoing infection. In the immunocompromised population, the REGN-COV2 cocktail, comprised of two noncompeting, neutralizing human IgG1 antibodies, is administered at 1200 mg dose every 3–4 weeks for optimal protection (61, 62). However, monoclonal antibodies have several limitations including high production cost and large dose requirements with frequent re-administration (63). Furthermore, acute *P. aeruginosa* infections progress rapidly and are difficult to treat due to increases in antibiotic resistance profiles (64). Previous works show that in acute infections, there is an extremely narrow therapeutic window for antibody administration prior to infection for protection (31, 65–67). Therefore, this study concludes Fc-engineering of an anti-Pseudomonal MAb can improve antibody effector function and highlights prophylactic delivery of these MAbs on DNA-plasmid as a promising approach to treat acute stage infections due to their durable pharmacokinetic profile (68, 69).

To date, there have been significant advances in the design and optimization of DMAbs in *in vivo* expression pharmacokinetics and functionality for numerous infectious disease models including their use in non-human primates (28, 34, 35, 70, 71). Recent findings show durable expression of DMAbs for 72 weeks post single-administration in humans (n=24/24) in a Phase I (NCT05293249) clinical trial with no anti-drug antibody (ADA) responses (72). In the current study, we demonstrate that Fc-point mutations can be strategically applied to modulate complement interactions and enhance anti-Pseudomonal DMAb efficacy. *In vitro*, we show that knocking out Fc-effector functions with the TM modification ablate V2L2-MD functionality, while incorporation of the 430G mutation we show that inclusion of the E430G Fc-modification significantly improves complement C1q binding and downstream complement deposition. Functional killing assays revealed that although both Fc-WT and Fc-E430G DMAbs mediate MAC-dependent bacterial killing, the E430G variant exhibited superior activity specifically via complement-mediated antibody-dependent cellular phagocytosis. This enhanced *in vitro* bactericidal effect translated to improve *in vivo* outcomes, as the Fc-E430G V2L2-MD DMAb showed trends in better control of bacterial load in a lethal acute pneumonia model against the virulent PAO1 strain in mice. Additionally, we demonstrate sustained DMAb expression for approximately 6-months post-single administration, supporting the feasibility of MAb delivery via synthetic DNA plasmid to bypass frequent re-administration of recombinant MAb therapies.

To improve treatment and protection against AMR pathogens, we previously showed a synergistic effect of anti-*Pseduomonas* DMAb and antibiotic as a strategy to reduce antibiotic usage (15). To build on this work, DMAbs with the E430G Fc-modification may not only improve DMAb potency in these instances but also be advantageous for immunocompromised patients at high-risk for *P. aeruginosa* infections that are often on long-term antibiotic treatment. This would include patients with cystic fibrosis, cancer, or hospitalized with burn wounds or undergoing organ transplants. In the current study, were only able to evaluate the prophylactic use of V2L2-MD DMAbs and could not evaluate the therapeutic properties between the Fc-WT and Fc-E430G variants in more detail. These constraints are largely attributable to the use of a rapid-onset, acute challenge model, which offers a narrow window of time to assess antibody-mediated protection. Moving forward, inclusion of longer or clinically relevant infection models would be beneficial to further characterize the therapeutic potential of Fc-modified DMAbs, including impact on inflammatory responses, bacterial dissemination, and survival. Here we also test DMAb efficacy against two laboratory reference strains PAO1 and PA14. While literature shows these strains differ in virulence and certain effector toxins, there are no observed differences in the PcrV binding epitope of V2L2-MD to either strain (18, 48). However, expanding *in vitro* killing assays to include a wider range of clinically relevant *P. aeruginosa* strains would be valuable to further characterize the breadth of activity and translational relevance of anti-Pseudomonal DMAbs. Taken together, our work expands on previous studies further demonstrating that utilization of complement-enhancing Fc-modification E430G can improve anti-PcrV DMAb effector function for improved outcomes during fatal acute *P. aeruginosa* challenge.

Antibody therapies have become a leading treatment for a range of human diseases with approximately 100 MAbs FDA approved for use in the United States. More importantly, MAbs have been shown to be safe and effective for use in immunocompromised hosts (10, 73). With on-going challenges in the development of successful vaccines and new antibiotics against *Pseudomonas aeruginosa* infections, mAb therapies are a promising treatment for high-risk, immunocompromised patients like those with Cystic Fibrosis or cancer. Combining advances in durable expression of DMAbs in humans with Fc-engineering strategies is a promising strategy to treat and prevent future *P. aeruginosa* infections in such high-risk populations. Our studies demonstrate the durability of V2L2-MD MAb when expressed in DNA plasmid. For the first time, we show that inclusion of the E430G Fc-modification significantly improves V2L2-MD MAb protection during an acute PAO1 challenge model in mice with a 1.5-log reduction of bacterial load in the lung and nasal wash, including dose sparing improvements in potency compared to Fc-WT. Together these studies support gene-delivered antibody development and Fc-engineering as an additional strategy to combat AMR infections, particularly against ESKAPE or Group D pathogens, where no successful vaccines are in development.

## Data Availability

The raw data supporting the conclusions of this article will be made available by the authors, without undue reservation.

## Supplementary material

Supplementary Figure 1 PA14 and PAO1 ADCP and ADCD killing assays. **(A)** PAO1 antibody-dependent cellular phagocytic killing assay (ADCP; no complement added). **(B)** PAO1 antibody-dependent complement deposition killing assay (ADCD; no phagocytes added). **(C)** PA14 antibody-dependent cellular phagocytic killing assay (ADCP; no complement added). **(D)** PA14 antibody-dependent complement deposition killing assay (ADCD; no phagocytes added). All killing assays utilized purified V2L2-MD DMAb variants at 200 µg dose. Percent (%) bacterial killing was calculated as ((mean of control - # DMAB variant colonies)/mean control) *100 (n=3/group). Negative percent bacterial killing represents bacterial growth. Statistical analysis performed in GraphPad Prism 10 by one-way ANOVA (* *p<0.05*, ** *p<0.01*, *** *p<0.001*, **** *p<0.0001*).

Supplementary Figure 2 Fold Change of PAO1 Bacterial Load in the Lung of all animals. Graph showing the fold change in lung bacterial load of all animals relative to average bacterial load in the Fc-WT DMAb group. (*) Asteriks denote outliers identified using GraphPad Prism 10 (Q=1%). Area under the curve analysis performed excluding outliers.

Supplementary Figure 3 Fold Change of PAO1 Bacterial Load in the Nasal Wash of all animals. Graph showing the fold change nasal wash bacterial load of all animals relative to average bacterial load in the Fc-WT DMAb group. (*) Asterisks denote outliers identified using GraphPad Prism 10 (Q=1%). Area under the curve analysis performed excluding outliers.
