# Supplementary material for: Fc-modification of anti-PcrV gene-encoded antibodies modulates complement-mediated killing of Pseudomonas aeruginosa
Source: Front Immunol. 2025 Jul 31;16:1618297. doi: 10.3389/fimmu.2025.1618297 (PMC12351402; doi:10.3389/fimmu.2025.1618297)
Supplement: Supplementary Figure 1 — PA14 and PAO1 ADCP and ADCD killing assays. (A) PAO1 antibody-dependent cellular phagocytic killing assay (ADCP; no complement added). (B) PAO1 antibody-dependent complement deposition killing assay (ADCD; no phagocytes added). (C) PA14 antibody-dependent cellular phagocytic killing assay (ADCP; no complement added). (D) PA14 antibody-dependent complement deposition killing assay (ADCD; no phagocytes added). All killing assays utilized purified V2L2-MD DMAb variants at 200 µg dose. Percent (%) bacterial killing was calculated as ((mean of control - # DMAB variant colonies)/mean control) *100 (n=3/group). Negative percent bacterial killing represents bacterial growth. Statistical analysis performed in GraphPad Prism 10 by one-way ANOVA (* p<0.05, ** p<0.01, *** p<0.001, **** p<0.0001). [file DataSheet1.docx]

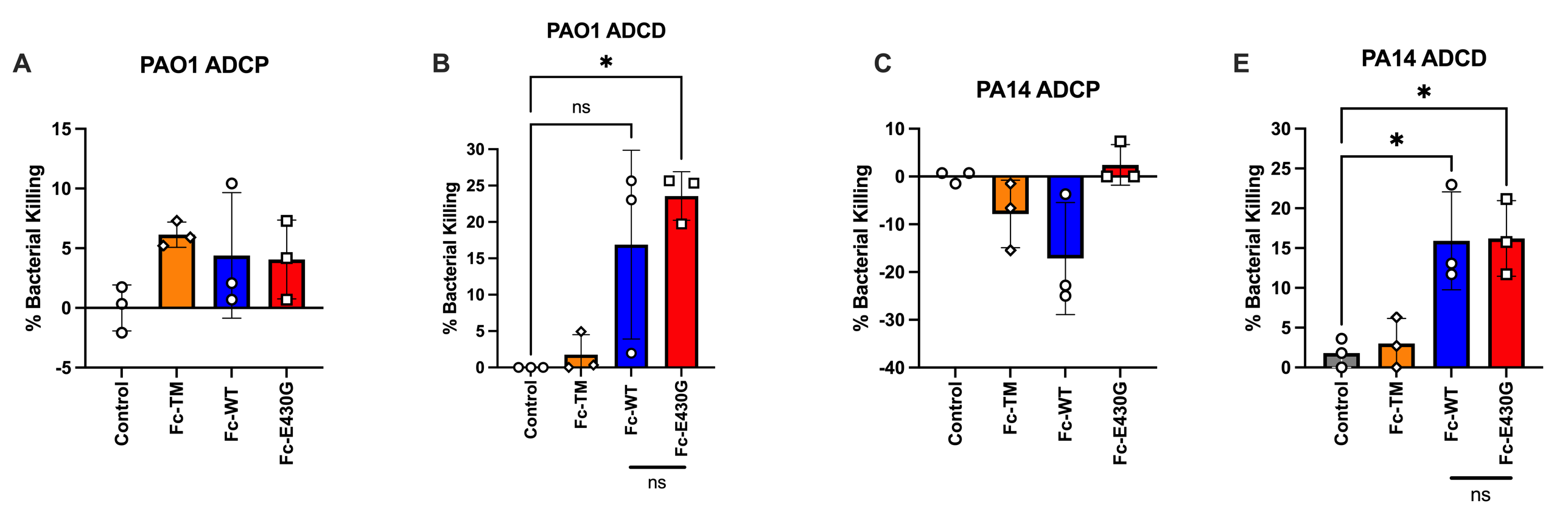


**Supplementary Figure 1: PA14 and PAO1 ADCP and ADCD killing assays. A)** PAO1 antibody-dependent cellular phagocytic killing assay (ADCP; no complement added). **B)** PAO1 antibody-dependent complement deposition killing assay (ADCD; no phagocytes added). **C)** PA14 antibody-dependent cellular phagocytic killing assay (ADCP; no complement added). **D)** PA14 antibody-dependent complement deposition killing assay (ADCD; no phagocytes added). All killing assays utilized purified V2L2-MD DMAb variants at 200 µg dose. Percent (%) bacterial killing was calculated as ((mean of control - # DMAB variant colonies)/mean control) *100 (n=3/group). Negative percent bacterial killing represents bacterial growth. Statistical analysis performed in GraphPad Prism 10 by one-way ANOVA (* *p<0.05*, ** *p<0.01*, *** *p<0.001*, **** *p<0.0001*).


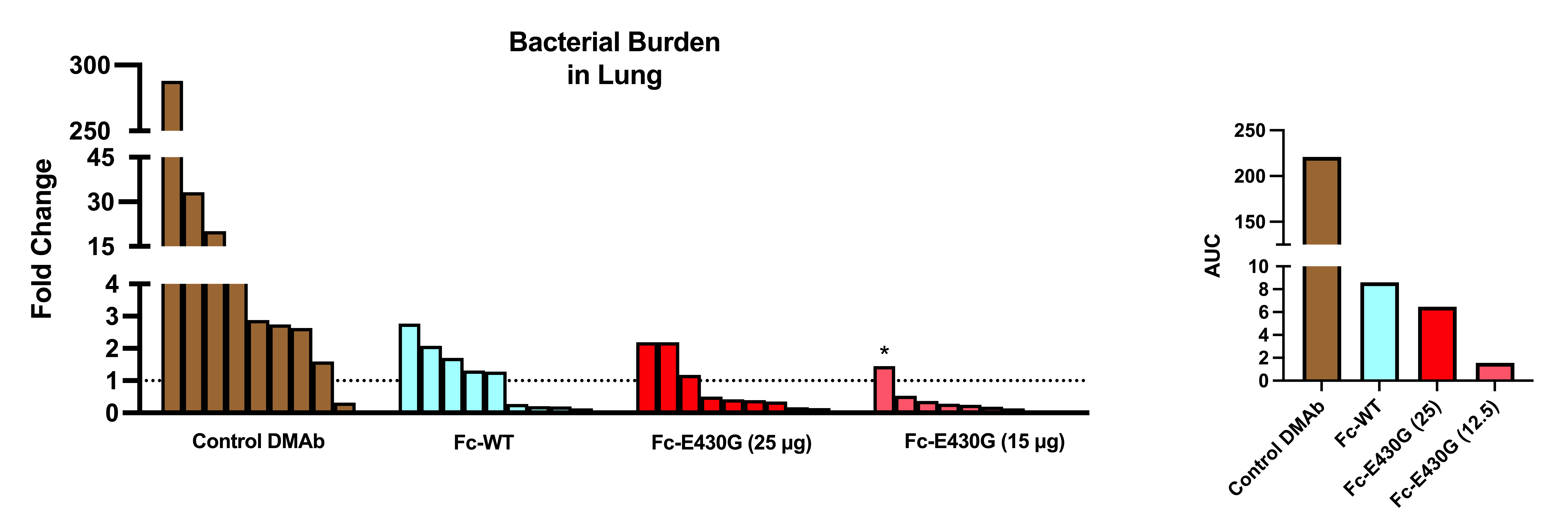


**Supplementary Figure 2: Fold Change of PAO1 Bacterial Load in the Lung of all animals.** Graph showing the fold change in lung bacterial load of all animals relative to average bacterial load in the Fc-WT DMAb group. (*) Asteriks denote outliers identified using GraphPad Prism 10 (Q=1%). Area under the curve analysis performed excluding outliers.


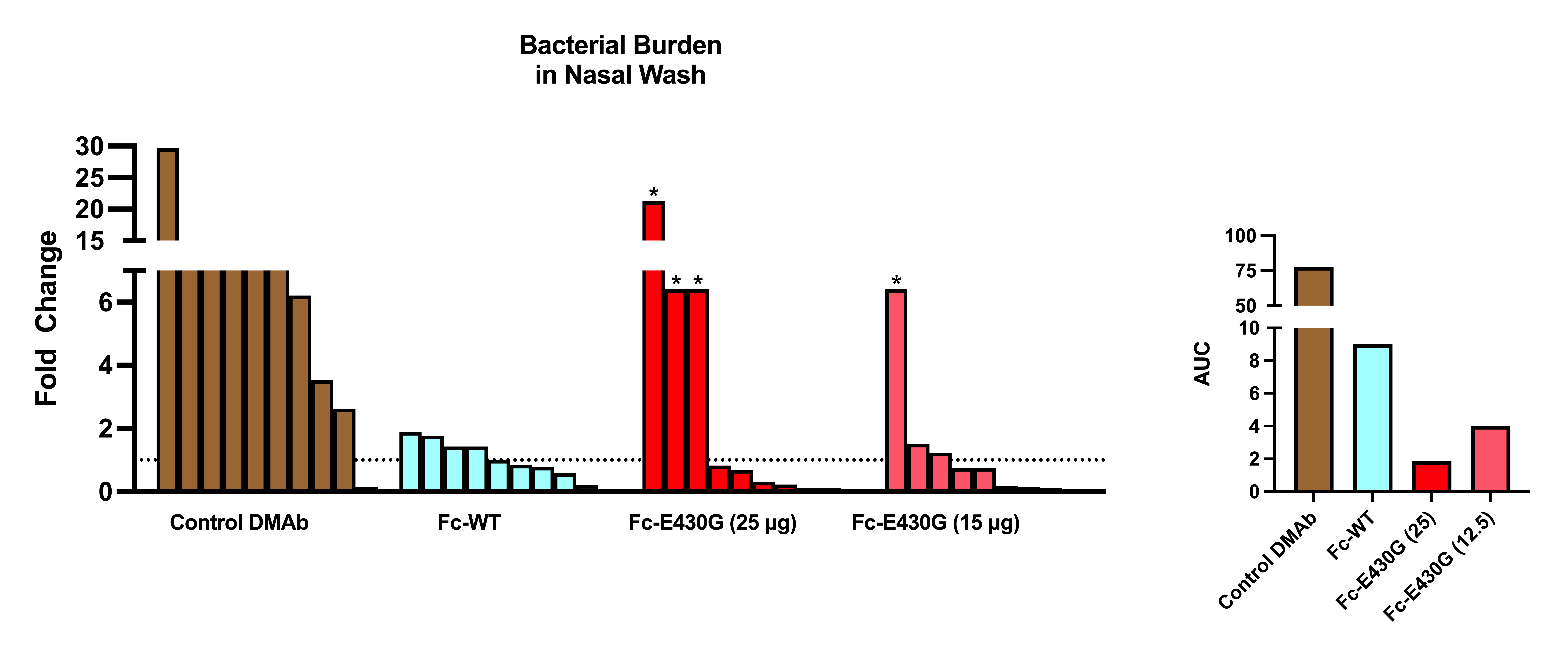


**Supplementary Figure 3: Fold Change of PAO1 Bacterial Load in the Nasal Wash of all animals.** Graph showing the fold change nasal wash bacterial load of all animals relative to average bacterial load in the Fc-WT DMAb group. (*) Asterisks denote outliers identified using GraphPad Prism 10 (Q=1%). Area under the curve analysis performed excluding outliers.
